# New Terpenes from the Egyptian Soft Coral *Sarcophyton ehrenbergi*

**DOI:** 10.3390/md12041977

**Published:** 2014-04-02

**Authors:** Ahmed Elkhateeb, Ahmed A. El-Beih, Amira M. Gamal-Eldeen, Montaser A. Alhammady, Shinji Ohta, Paul W. Paré, Mohamed-Elamir F. Hegazy

**Affiliations:** 1 Phytochemistry and Plant Systematic Department, National Research Centre, El-Tahrir St., Dokki, Giza 12311, Egypt; E-Mail: chem_ahmed@hotmail.com; 2 Department of Chemistry of Natural & Microbial Products and Center of Excellence Advanced Sciences, National Research Centre, El-Tahrir Street, Dokki, Giza 12311, Egypt; E-Mail: aae2eg@yahoo.com; 3 Cancer Biology Lab, Center of Excellence for Advanced Sciences & Biochemistry Department, National Research Center, Dokki, Cairo 12311, Egypt; E-Mail: aeldeen7@yahoo.com; 4 National Institute of Oceanography and Fisheries, Red Sea Branch, Hurghada 84511, Egypt; E-Mail: coralreef_niof1@yahoo.com; 5 Graduate School of Biosphere Science, Hiroshima University, 1-7-1 Kagamiyama, Higashi-Hiroshima 739-852, Japan; E-Mail: ohta@hiroshima-u.ac.jp; 6 Department of Chemistry & Biochemistry, Texas Tech University, Lubbock, TX 79409, USA; E-Mail: paul.pare@ttu.edu; 7 Chemistry of Medicinal Plants Department and Center of Excellence Advanced Sciences, National Research Centre, El-Tahrir Street, Dokki, Giza 12311, Egypt

**Keywords:** *Sarcophyton ehrenbergi*, soft coral, terpenes, cembranoids, sesquiterpenes, cytotoxic activity

## Abstract

Chemical investigations of the Egyptian soft coral *Sarcophyton ehrenbergi* have led to the isolation of compounds **1**–**3** as well as the previously reported marine cembranoid diterpene sarcophine (**4**). Structures were elucidated by comprehensive NMR and HRMS experimentation. Isolated compounds were *in vitro* assayed for cytotoxic activity against human hepatocarcinoma (HepG2) and breast adenocarcinoma (MCF-7) cell lines.

## 1. Introduction

**Figure 1:**
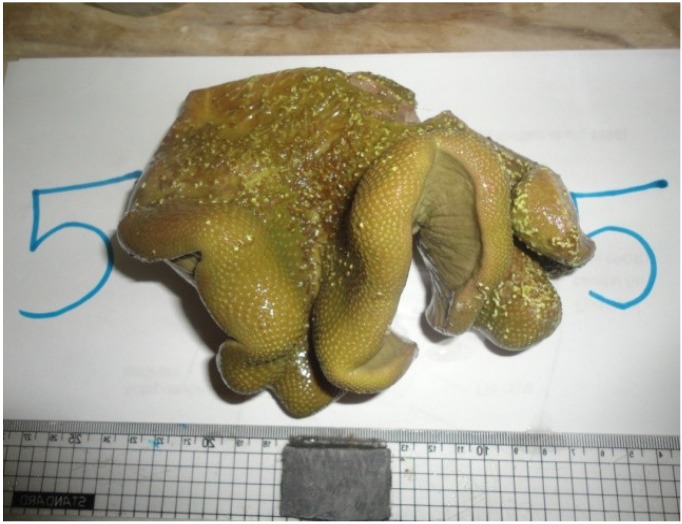
Soft coral *Sarcophyton ehrenbergi*.

**Figure 2:**
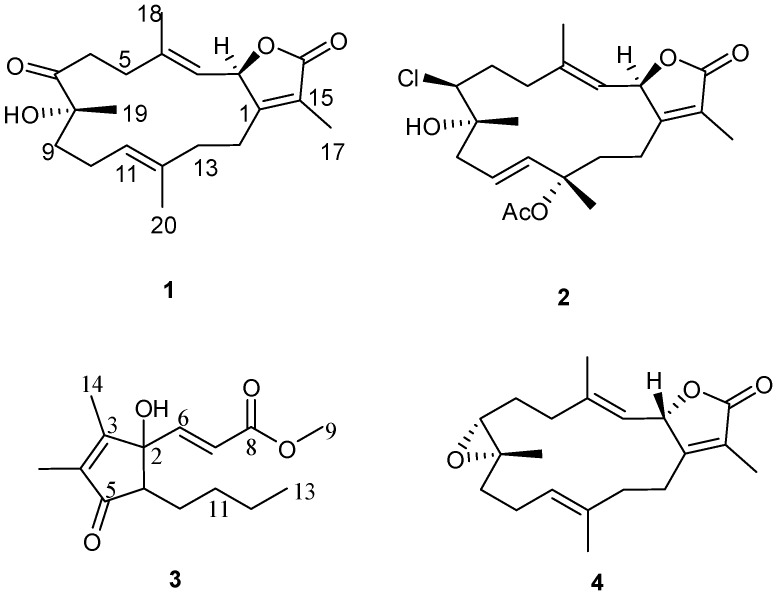
Structures of metabolites **1**–**4.**

The Red Sea serves as an epicenter for marine bio-diversity, with a high endemic biota. Indeed, of the 180 soft coral species identified world-wide, approximately 40% are native to the Red Sea [1]. Soft coral (Cnidaria: Anthozoa: Octocorallia) are an important structural component of coral reef communities contributing significantly to coral-reef biomass [2,3]. Soft coral have also been the subject of biological studies since the 19th century with coral belonging to the genus *Sarcophyton* (Alcyoniidae) well recognized as a rich source of terpenoids [4]. For cembranoid-type diterpenes, a wide range of biological activities have been reported including antitumor, ichthyotoxic, anti-inflammatory, neuroprotective, antibacterial, antiangiogenic, antimetastatic, anti-osteoporotic, and cytotoxic properties [5]. Diterpenes have been isolated previously from *Sarcophyton ehrenbergi* [6,7,8,9,10]; however, to the best of our knowledge, this is the first chemical investigation of the Red Sea soft coral *Sarcophyton ehrenbergi* (Figure 1). In the course of our research for bioactive substances from marine sources [11,12], chromatographic separation of an ethyl acetate extract of the alcyonacean soft coral, *S. ehrenbergi* has led to the isolation of three compounds **1**–**3** as well as the previously reported marine cembranoid diterpene sarcophine (**4**) (Figure 2). Structures of these isolated metabolites were elucidated by comprehensive NMR and HRMS techniques. Identified compounds were *in vitro* assayed for cytotoxic activity in two human cancer cell lines.

## 2. Results and Discussion

The EtOAc extract of freshly collected *S. ehrenbergi* was subjected to normal and reverse phase chromatography to afford the isolated metabolites as reported. 

**Figure 3:**
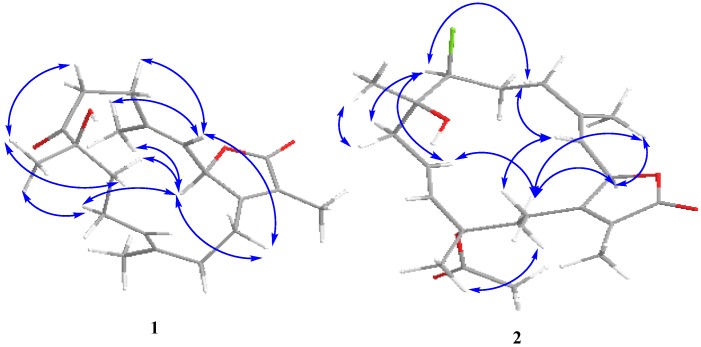
NOESY correlations for **1**–**2**.

Compound **1** was obtained as a colorless oil. The molecular formula C_20_H_28_O_4_, determined by HR-EI-MS (*m/z* 332.1935, [M]^+^, C_20_H_28_O_4_; calcd. 332.1988) points to seven degrees of unsaturation. NMR data were consistent with a sarchophine skeleton previously isolated from other *Sarcophyton* species [11,12] and reported here (**4**). Select ^13^C NMR signals were consistent with an α,β-unsaturated-γ-lactone functionality: δ 123.1 (C-15), δ 162.9 (C-1) δ 174.9 (C-16). Carbon NMR chemical shifts (Supplementary Figure S2) established olefinic carbons at δ_C_ 120.2/143.9 (C-3/C-4) and δ_C_ 123.9/135.2 (C-11/C-12), a ketone functionality at δ_C_ 213.5 (C-7) and oxygen functionalities at δ_C_ 79.4 (C-2) and δ_C_ 78.6 (C-8); all signals were consistent with a sarchophine skeleton. The olefin carbons at C-3 and C-11 were assumed to be in the *E* configuration based on other sarchophine derivatives [11,12,13]; this orientation was confirmed by NOESY correlations. The ^1^H, ^1^H COSY spectrum revealed the coupling of four hydrocarbon regions common to sarchophine skeletons: δ_H_ 5.47 (d, *J* = 10.3 Hz, H-2) and 4.96 (br. d, *J* = 10.3 Hz, H-3); δ_H_ 2.29 (m, H_2_-5) and 2.46 (m, H_2_-6); δ_H_ 1.87/1.98 (m, H_2_-9), 2.04/2.18 (m, H_2_-10) and 4.72 (br. t, *J* = 7.6 Hz, H-11) and δ_H_ 2.04 (m, H_2_-13) and 2.29/2.46 (m, H_2_-14). HMBC correlations (Supplementary Figure S4) between H-2 and δ_C_ 162.9 (C-1), H-3 and δ_C_ 31.5 (C-5), H_2_-5 and δ_C_ 143.9 (C-4) and H_2_-6/H_2_-5 and δ_C_ 213.5 (C-7) established the carbon linkages from C-1 to C-7. Additionally, correlations between H_2_-9 and δ_C_ 213.5 (C-7)/78.6 (C-8)/123.9 (C-11), H-11 and δ_C_ 35.9 (C-13), H_2_-13 and δ_C_ 135.2 (C-12)/162.9 (C-1) and H_2_-14 and δ_C_ 162.9 (C-1)/79.4 (C-2) completed connectivities of the 14-membered ring. The methyl groups’ positions were established by HMBC correlations between C-3, C-4 and C-5 and δ_H_ 1.94 (H_3_-18, br. s); C-7, C-8 and C-9 and δ_H_ 1.31 (H_3_-19, s); C-11, C-12 and C-13 and δ_H_ 1.56 (H_3_-20, br. s); and C-1, C-15 and C-16 and δ_H _1.81 (H_3_-17, s). The relative configuration of **1** was determined on the basis of coupling constants and NOESY experiments. The vicinal coupling constant of 10.3 Hz between H-2 and H-3 suggested a *cis* configuration between the γ-lactone proton (H-2) and the olefinic proton (H-3) which was confirmed by observed NOESY correlations (Figure 3). Correlations were observed between Me-19 and H-6 (δ_H_ 2.91) as well as between H-9 (δ_H_ 1.98) and H-10 (δ_H_ 2.04); additionally, H-2 (δ_H_ 5.47) showed a correlation with H-18 (δ_H_ 1.94) and H-10 (δ_H_ 2.18) which indicated that Me-19 and an OH group at C-8 (δ_C_ 78.6) were positioned in β and α configurations, respectively [11,12,13].

**Figure 4:**
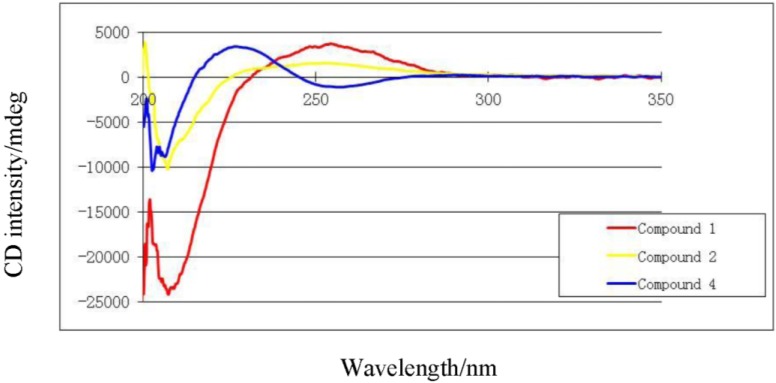
Circular dichroism spectra of compounds **1**, **2** and **4**.

The absolute stereochemistry of **1** at C-2 was determined via circular dichroism (CD) analysis (Figure 3). The observed positive Cotton effect ([θ]_255_ +4) followed by a negative value ([θ]_225_ −24.8) observed in the CD spectrum (Figure 4) for the electronic transitions of the 2(5*H*)-furanone moiety, indicated a left-handed (*M*) helix configuration for the five-membered α,β-unsaturated-γ-lactone ring [14]. CD spectral comparisons between **1** and sarcophine (**4**) show a shifted spectrum establishing an inverted stereochemistry at C-2 for **1** and **4** (Figure 4); with **4** having a literature assigned *S* absolute configuration, the absolute configuration for **1** at C-2 was confirmed to be *R* [12,14,15]. From the above evidence, the structure of **1** was confirmed to be 7-keto-8α-hydroxy-deepoxysarcophine (Figure 2).

**Figure 5:**
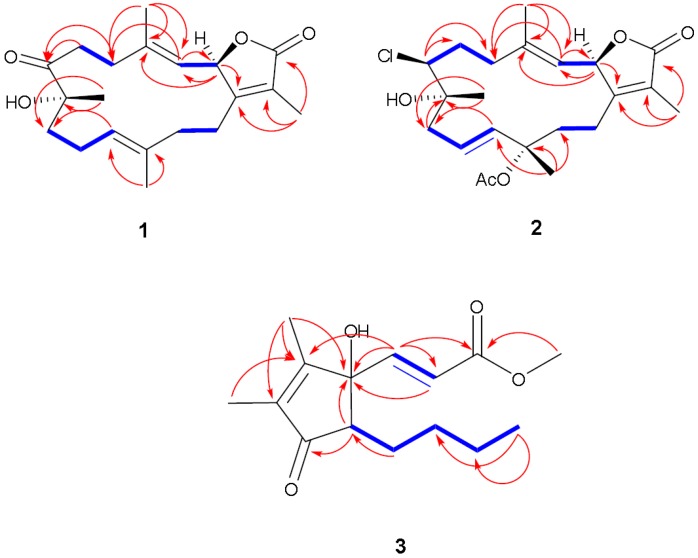
Selected ^1^H-^1^H COSY (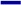) and HMBC (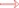) correlations of **1**–**3**.

Compound **2** was obtained as a colorless oil. The molecular formula C_22_H_32_ClO_5_ was determined by HR-FAB-MS (*m/z* 411.1943, [M + H]^+^, C_22_H_32_ClO_5_^+^; calcd. 411.1938) and was consistent with seven degrees of unsaturation. The NMR spectrum of **2** was quite similar to **1** except for several noted differences. First, there was a disappearance of the C-7 ketone signal accompanied by the appearance of an up-field signal at δ_C_ 62.9 (C-7). This C-7 carbon signal was coupled by HMQC (Supplementary Figure S7) with a downfield doublet signal at δ_H_ 3.45 (*J* = 11.0 Hz) consistent with a chlorine atom at C-7 [13,14,15]. HMBC correlations (Supplementary Figure S8) between δ_H_ 3.45 and δ_C_ 36.0 (C-5), 26.3 (C-6) and 74.8 (C-8) confirmed the proton signal assignment to H-7. Second, there was a downfield shift of C-20 by 9 ppm to δ_C_ 24.4 accompanied by the appearance of carbonyl and methyl groups at δ_C_ 170.0 and 22.2, respectively. These data were consistent with an acetoxy group at C-12 and the displacement of an olefinic bond between C-11 and C-12 (compound **1**) to a C-10-C-11 double bond position. This double bond shift was confirmed by COSY correlations starting from H_2_-9 (δ_H_ 2.20/2.62, m) correlating with δ_H_ 5.53 (H-10, ddd, *J* = 15.8, 10.3, 4.8 Hz) and δ_H_ 5.82 (H-11, d, *J* = 15.8 Hz). HMBC correlations were also observed between H_2_-9 and δ_C_ 138.2 (C-11) and H-20 and δ_C_ 138.2 (C-11), 82.0 (C-12) and 41.7 (C-13). HMBC correlations were also observed between the acetoxy methyl signal (δ_C_ 2.05) and the carbonyl ester (δ_C_ 170) as shown in Figure 5 (Supplementary Figure S8). The relative configuration of **2** was determined on the basis of coupling constants, and NOESY experiments. The vicinal coupling constant of 10.3 Hz between H-2 and H-3 suggested a *cis* configuration between the γ-lactone proton (H-2) and the olefinic proton (H-3) that was confirmed by observed NOESY correlations (Figure 3). The NOE correlations between H-7 and H-10 (δH 5.53), H-9 (δH 2.20) and Me-18 (δH 1.76), Me-18 and H-2 (δH 5.42) and Me-19 and H-9 (δH 2.62), indicate a β chlorine conformation at C-7 (δC 62.9). In addition, the NOE interactions between H-2 and H-14 (δH 2.20) and H-14 (δH 2.62) and Me-20 (δH 1.63) reveal an α orientation for the acetoxy group at C-12. With nearly equivalent CD spectra observed for **2** compared with **1** (Figure 4) assignment of an (*R*) configuration at C-2 as well as absolute stereochemistry at the other stereo centers was possible [12,14,15]. From the above spectral data, **2** was identified as 7β-chloro-8α-hydroxy-12-acetoxy-deepoxysarcophine (Figure 2).

Compound **3** was obtained as a colorless oil. The molecular formula C_15_H_22_O_4_ was determined by HR-EI-MS (*m/z* 266.1552, [M]^+^, C_15_H_22_O_4_; calcd. 266.1518) and consistent with five degrees of unsaturation. Starting with a carbonyl carbon based on a chemical shift of δ_C_ 204.7 (C-5) and HMBC data (Supplementary Figure S12), a cyclopentenone ring structure with two methyl groups was deduced. Informative correlations were observed between C-5 and δ_H_ 1.73 (H_3_-15)/2.42 (H-1); H-1 and δ_C_ 82.8 (C-2); C-2 and δ_H_ 1.87 (H_3_-14); H_3_-14 and δ_C_ 165.4 (C-3)/137.5 (C-4), C-3 and δ_H_ 1.73 (H_3_-15) and C-4 and H_3_-15. HMBC data established that the pentone ring was linked to olefinic protons via C-2 with correlations observed between C-2 and δ_H_ 6.60 (H-6, d, *J* = 15.8 Hz) and C-2 and δ_H_ 6.21 (H-7, d, *J* = 15.8 Hz) with the olefin in a *Z* configuration based on the coupling constant. The methyl ester terminus was linked to an olefinic group through HMBC correlations between H-6 and δ_C_ 166.4 (C-8) as well as between C-8 and H-7. From COSY correlations, a four carbon unbranched saturated hydrocarbon originating from C-1 was identified. The H-1 NMR proton signal correlated with δ_H_ 1.30 (H-10, m)/1.82 (H-10, m); H-10 correlated with δ_H_ 1.32 (H-11, m)/1.45 (H-11, m); H-11 correlated with δ_H_ 1.30 (H-12, m); and H-12 correlated with δ_H _0.87 (H-13, t, *J* = 7.6 Hz). The relative structure of **3** was elucidated by an analysis of coupling constant. The coupling constant of H-1 (δ 2.42, dd) of 8.9 and 5.5 Hz indicated that H-1 is on the β-face. From the above evidence, the structure of **3** was assigned to be (*E*)-methyl-3-(5-butyl-1-hydroxy-2,3-dimethyl-4-oxocyclopent-2-enyl) acrylate.

Using a 3-[4,5-dimethylthiazole-2-yl]-2,5-diphenyltetrazolium bromide (MTT) spectrophotometric cell-viability assay, the biological activity of the reported coral metabolites to lower human hepatocarcinoma (HepG2) and breast adenocarcinoma (MCF-7) cell-line viability was assayed. The treatment of Hep-G2 cells with metabolites **1**–**4** (100–12.5 μg/mL) did not exhibit a measureable cytotoxic effect. Metabolites **1**–**3** exhibited a moderate cytotoxic effect with the MCF-7 cell-line with IC_50_ values of 192.87, 68.57, and 114.41 μmol/mL, respectively.

## 3. Experimental Section

### 3.1. General Experimental Procedures

^1^H and ^13^C NMR spectra were recorded in CDCl_3_ on a JEOL ECA-600 spectrometer (600 MHz for ^1^H and 150 MHz for ^13^C, respectively). All chemical shifts (δ) are given in ppm units with reference to TMS as an internal standard and coupling constants (*J*) are reported in Hz. FAB-MS was performed on a Finnigan LCQ ion trap mass spectrometer and HR-FAB-MS experiments were performed on Fourier transform ion cyclotron mass spectrometer (Ion Spec, Varian, Walnut Creek, CA, USA). EI-MS experiments were performed using a Thermo ISQ Single Quadrupole system (Thermo Scientific, San Jose, CA, USA). High performance liquid chromatography (HPLC) was performed on an Agilent pump equipped with an Agilent-G1314 variable wavelength UV detector at 254 nm and a semi-preparative reverse-phase column (Econosphere™, RP-C18, 5 μm, 250 × 4.6 mm, Alltech, Deerfield, IL, USA). Optical rotation was determined at 589 nm (sodium D line) using a Perkin–Elmer-341 MC digital polarimeter (Wellesley, MA, USA); [α] D-values are given in units of 10 deg^−1^·cm^2 ^g^−1^. CD was measured with an OLIS, DSM-10 UV/Vis CD (Olis, Bogart, GA, US). Silica gel 60 (230–400 mesh, Merck, Darmstadt, Germany) and Sephadex LH-20 (Sigma, St. Louis, MO, USA) were used for column chromatography. Pre-coated silica gel plates (Merck, Darmstadt, Germany, Kieselgel 60 F_254_, 0.25 mm) were used for TLC analyses. Spots were visualized by heating after spraying with 10% H_2_SO_4_.

### 3.2. Animal Material

Soft coral *S. ehrenbergi* was collected from the Egyptian Red Sea off the coast of Hurghada in March 2012. The soft coral was identified by co-author Alhammady with a voucher specimen (03RS27) deposited in the National Institute of Oceanography and Fisheries, Marine Biological Station, Hurghada, Egypt.

**Table 1:** ^1^H and ^13^C NMR spectral data of **1**–**3** ^a^.

Position	1		2		3	
	δ_H _(*J* in Hz)	δ_C_	δ_H _(*J* in Hz)	δ_C_	δ_H _(*J* in Hz)	δ_C_
1	−	162.9 C	−	161.9 C	2.42 (dd, 8.9, 5.5)	60.0 CH
2	5.47 (d, 10.3)	79.4 CH	5.42 (d, 10.3)	78.3 CH	−	82.8 C
3	4.96 (br d, 10.3)	120.2 CH	4.98 (d, 10.3)	122.2 CH	−	165.4 C
4	−	143.9 C	−	142.9 C	−	137.5 C
5	2.29 (m) 2.46 (m)	31.5 CH_2_	2.26 (m) 2.42 (m)	36.0 CH_2_	−	204.7 C
6	2.81 (m) 2.91 (m)	34.6 CH_2_	1.63 (m) 2.20 (m)	26.3 CH_2_	6.60 (d, 15.8)	148.2 CH
7	−	213.5 C	3.45 (d, 11.0)	62.9 CH	6.21 (d, 15.8)	121.5 CH
8	−	78.6 C	−	74.8 C	−	166.4 C
9	1.87 (m) 1.98 (m)	39.5 CH_2_	2.20 (m) 2.62 (m)	43.3 CH_2_	3.74 (s)	51.9 CH_3_
10	2.04 (m) 2.18 (m)	22.0 CH_2_	5.53 (ddd, 15.8, 10.3, 4.8)	123.1 CH	1.30 (m) 1.82 (m)	25.4 CH_2_
11	4.72 (br t, 7.6)	123.9 CH	5.82 (d, 15.8)	138.2 CH	1.32 (m) 1.45 (m)	30.1 CH_2_
12	−	135.2 C	−	82.0 C	1.30 (m)	22.7 CH_2_
13	2.04 (m)	35.9 CH_2_	2.10 (m) 1.38 (m)	41.7 CH_2_	0.87 (t, 7.6)	13.9 CH_3_
14	2.29 (m) 2.46 (m)	25.7 CH_2_	2.20 (m) 2.62 (m)	21.5 CH_2_	1.87 (s)	11.0 CH_3_
15	−	123.1 C	−	123.5 C	1.73 (br s)	8.5 CH_3_
16	−	174.9 C	−	175.0 C		
17	1.81 (s)	9.0 CH_3_	1.84 (s)	8.9 CH_3_		
18	1.94 (br s)	18.3 CH_3_	1.76 (br s)	15.2 CH_3_		
19	1.31 (s)	29.0 CH_3_	1.32 (s)	23.7 CH_3_		
20	1.56 (br s)	15.6 CH_3_	1.63 (s)	24.4 CH_3_		
21			−	170.0 C		
22			2.05 (s)	22.2 CH_3_		

^a^ Recorded in CDCl_3_ and obtained at 600 and 150 MHz for ^1^H and ^13^C NMR, respectively.

### 3.3. Extraction and Separation

Frozen soft coral (4 kg, total wet weight) was chopped into small pieces and extracted with ethyl acetate at room temperature (4 L × 5). The combined ethyl acetate extracts were concentrated *in vacuo* to a brown gum. The dried EtOAc-soluble material (250 g) was subjected to gravity chromatography in a silica gel column (6 × 120 cm) using *n*-hexane-EtOAc gradient separated into eight fractions.

Fraction 4 (2.2 g) eluted with *n*-hexane–EtOAc (6:1) was subjected to silica gel column separation to afford **4** (100 mg). The remaining sub-fraction 4 was collected and re-purified by HPLC using MeOH/H_2_O (8:2 v/v) to afford **3** (15 mg). Fraction 5 (75.3 mg) was purified by Sephadex LH-20 using hexane-CHCl_3_-MeOH (7:4:0.5) followed by reverse phase HPLC in MeOH/H_2_O (7:3 v/v) to afford **1** (17 mg), and **2** (20 mg).

7-Keto-8α-hydroxy-deepoxysarcophine (**1**): Colorless oil; 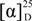 = +15.0 (*c* 0.03, CHCl_3_); ^1^H NMR and ^13^C NMR data, see Table 1; HR-EI-MS [M]^+^ *m/z* 332.1935 (calc. 332.1988, C_20_H_28_O_4_).

7β-Chloro-8α-hydroxy-12-acetoxy-deepoxysarcophine (**2**): Colorless oil; 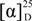 = −20.1 (*c* 0.01, CHCl_3_); ^1^H NMR and ^13^C NMR data, see Table 1; HR-FAB-MS [M + H]^+^ *m/z* 411.1943 (calc. 411.1938, C_22_H_32_ClO_5_).

(*E*)-Methyl-3-(5-butyl-1-hydroxy-2,3-dimethyl-4-oxocyclopent-2-enyl)acrylate(**3**): Colorless oil; 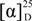 = +1.2 (*c* 0.01, CHCl_3_); ^1^H NMR and ^13^C NMR data, see Table 1; HR-EI-MS [M]^+^ *m/z* 266.1552 (calc. 266.1518 C_15_H_22_O_4_).

(+)-Sarcophine (**4**): 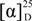 = +95.0 (*c* 0.5, CHCl_3_); lit. 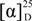 = +92 (*c* 1.0, CHCl_3_) [15].

### 3.4. Cell Culture

Cell-culture material was purchased from Cambrex BioScience (Copenhagen, Denmark) and chemicals from Sigma-Aldrich (St. Louis, MO, USA), except where noted otherwise. Human hepatocarcinoma and breast adenocarcinoma cell lines, HepG2 and MCF-7 respectively, purchased from the American type culture collection (ATCC, Manassas, VA, USA) were used to evaluate the cytotoxic effect of the isolated metabolites. Cells are routinely cultured in Dulbecco’s modified eagle’s medium (DMEM) supplemented with 10% fetal bovine serum (FBS), 2 mM l-glutamine, 100 units/mL penicillin G sodium, 100 units/mL streptomycin sulphate and 250 ng/mL amphotericin B. Cells are maintained at sub-confluency in humidified air at 37 °C containing 5% CO_2_. For sub-culturing, monolayer cells are harvested after trypsin/EDTA treatment. Cells for bioassays were assayed at 75% confluence. Test compounds were dissolved in dimethyl sulphoxide (DMSO) and diluted to begin with a concentration of 100 μg/mL and serially diluted to a final concentration of 12.5 μg/mL. Assays were run in triplicate unless noted otherwise.

### 3.5. Anti-Tumor Activity

Compound cytotoxicity against HepG2 and MCF-7 cell lines was assayed via a 3-[4,5-dimethylthiazole-2-yl]-2,5-diphenyltetrazolium bromide (MTT) spectrophotometric cell-viability assay. The MTT assay is based on the ability of active mitochondrial dehydrogenase enzyme of living cells to cleave the tetrazolium ring of the yellow MTT to form dark-blue insoluble formazan crystals that are largely impermeable to cell membranes, resulting in its accumulation within healthy cells. With loss of cell viability, formazan crystals leak into the media. Cell viability is monitored and quantified spectrophotometrically from formazan crystals extracted from the cells [16]. Briefly, cells are diluted in serum-free media to 5.0 × 10^4^ cells/well based on turbidity measurements, plated into flat-bottom 96-well microplates, and incubated for 48 h with 20 μL of test sample. After incubation, media is removed and 40 μL MTT (0.5 mg MTT/ ml 0.9% aqueous NaCl) is added into each well and then incubated for an additional 4 h. MTT crystals are solubilized by adding 180 µL of acidified isopropanol to each well and plates are agitated at room temperature, followed by absorbance measurements at 570 nm using a microplate ELISA reader (BMG LABTECH, Ortenberg, Germany). Triplicate repeats were performed for each concentration and the average was calculated. Data were expressed as the percentage of relative viability compared with the untreated cells and with the vehicle control. Cytotoxicity was reported as a relative viability compared with the untreated control. Percent relative viability was determined based cell viability: (Absorbance of treated cells/absorbance of control cells) × 100. Half maximal inhibitory concentrations (IC_50_) were calculated based on dose-response curves.

## 4. Conclusions

Three new (**1**–**3**) and one previously reported (**4**) terpenes were isolated and chemically characterized from the Red Sea soft coral *S.ehrenbergi*. Identified compounds **1**–**3** exhibited *in vitro* cytotoxic activity against a breast adenocarcinoma cell line (MCF-7) with IC_50_ values of 192.87, 68.57, and 114.41 µm/mL, respectively.

## Supplementary Files

Supplementary File 1 Supplementary Information (PDF, 1002 KB)
