# Supplementary material for: New Terpenes from the Egyptian Soft Coral Sarcophyton ehrenbergi
Source: Mar Drugs. 2014 Apr 2;12(4):1977–86. doi: 10.3390/md12041977 (PMC4012453; doi:10.3390/md12041977)
Supplement: Supplementary File 1 — Supplementary Information (PDF, 1002 KB) [file marinedrugs-12-01977-s001.pdf]

Supplementary Information

Figure S1. ^{1}H NMR spectrum of 7 Keto, 8 hydroxy sarcophine (**1**) in $CDCl_3$.
Figure S2. ^{13}C NMR spectrum of 7 Keto, 8 hydroxy sarcophine (**1**) in $CDCl_3$.
Figure S3. HMBC spectrum of 7 Keto, 8 hydroxy sarcophine (**1**) in $CDCl_3$.
Figure S4. HMBC spectrum of 7 Keto, 8 hydroxy sarcophine (**1**) in $CDCl_3$.
Figure S5. ^{1}H NMR spectrum of 7β-Chloro-8α-hydroxy-12-acetoxy-deepoxysarcophine (**2**) in $CDCl_3$.
Figure S6. ^{13}C NMR spectrum of 7β-Chloro-8α-hydroxy-12-acetoxy-deepoxysarcophine (**2**) in $CDCl_3$.
Figure S7. HMQC spectrum of 7β-Chloro-8α-hydroxy-12-acetoxy-deepoxysarcophine (**2**) in $CDCl_3$.
Figure S8. HMBC spectrum of 7β-Chloro-8α-hydroxy-12-acetoxy-deepoxysarcophine (**2**) in CDCl3.
Figure S9. ^{1}H NMR spectrum of (*E*)-Methyl-3-(5-butyl-1-hydroxy-2,3-dimethyl-4-oxocyclopent-2-enyl)acrylate (**3**) in $CDCl_3$.
Figure S10. ^{13}C NMR spectrum of (*E*)-Methyl-3-(5-butyl-1-hydroxy-2,3-dimethyl-4-oxocyclopent-2-enyl)acrylate (**3**) in $CDCl_3$.
Figure S11. HMQC spectrum of (*E*)-Methyl-3-(5-butyl-1-hydroxy-2,3-dimethyl-4-oxocyclopent-2-enyl)acrylate (**3**) in $CDCl_3$.
Figure S12. HMBC spectrum of (*E*)-Methyl-3-(5-butyl-1-hydroxy-2,3-dimethyl-4-oxocyclopent-2-enyl)acrylate (**3**) in $CDCl_3$.

Figure S1. ^{1}H NMR spectrum of 7 Keto, 8 hydroxy sarcophine (**1**) in $CDCl_3$.

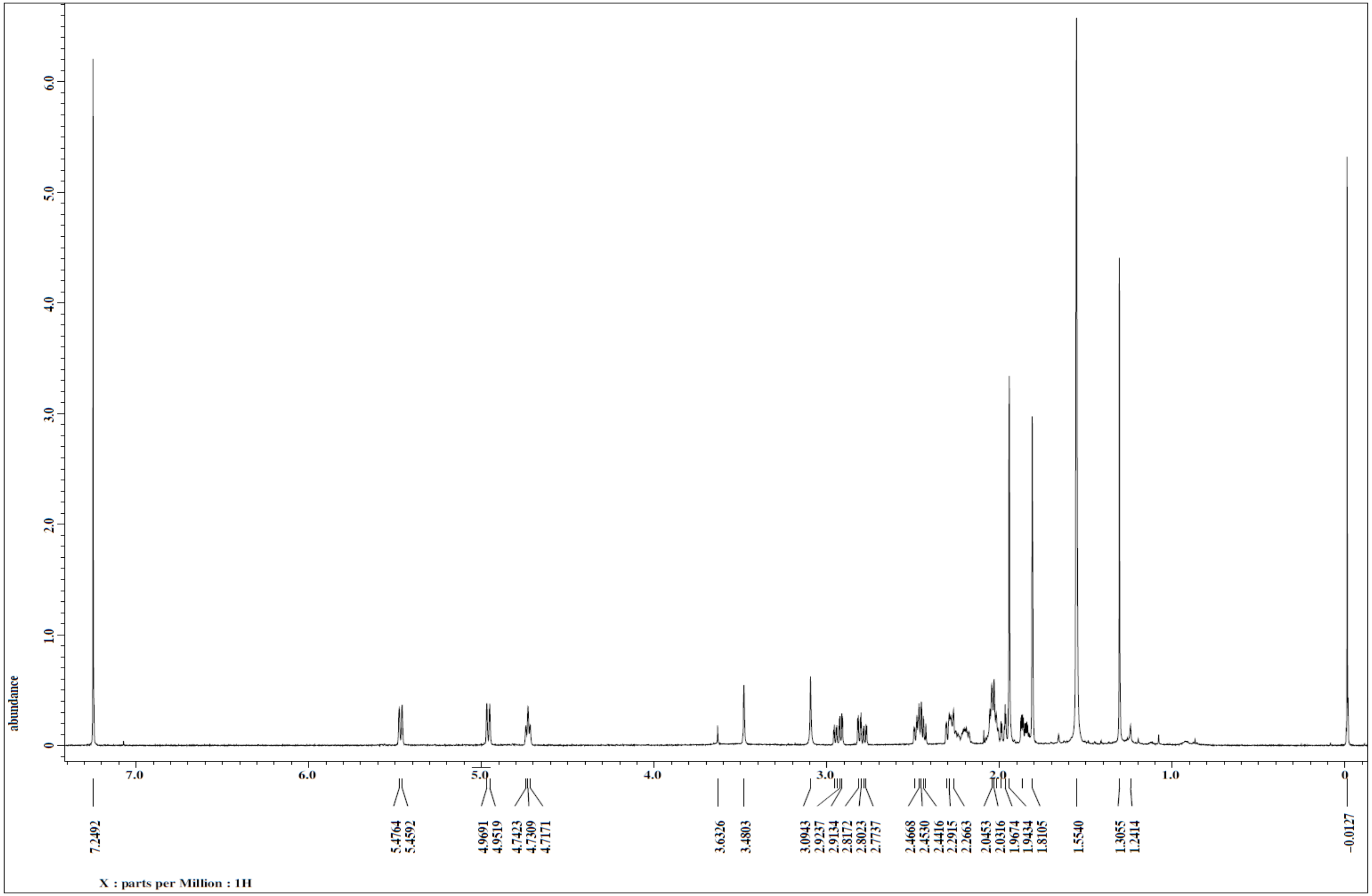

Figure S2. ^{13}C NMR spectrum of 7 Keto, 8 hydroxy sarcophine (**1**) in $CDCl_3$.

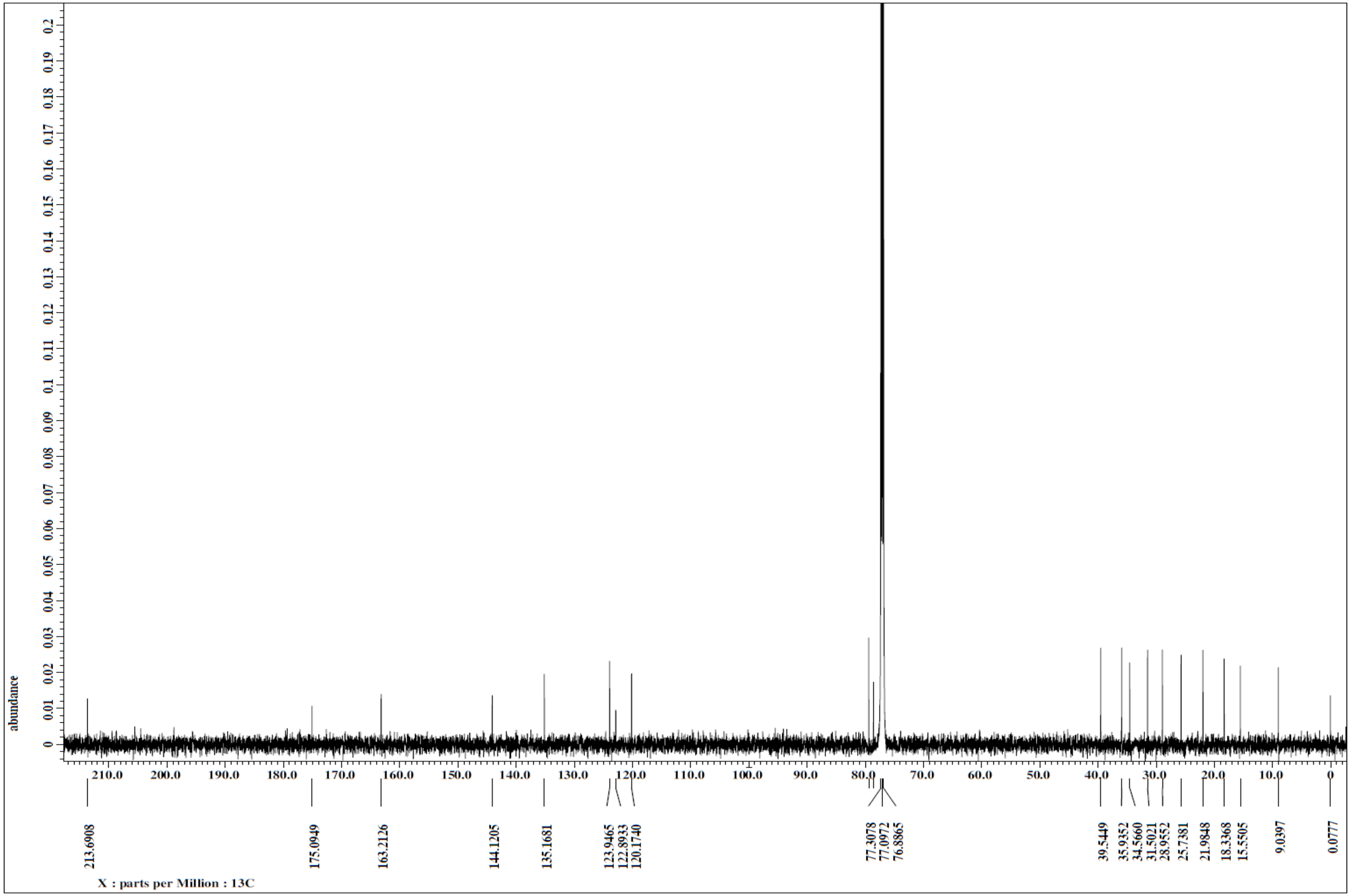

Figure S3. HMQC spectrum of 7 Keto, 8 hydroxy sarcophine (**1**) in $CDCl_3$.

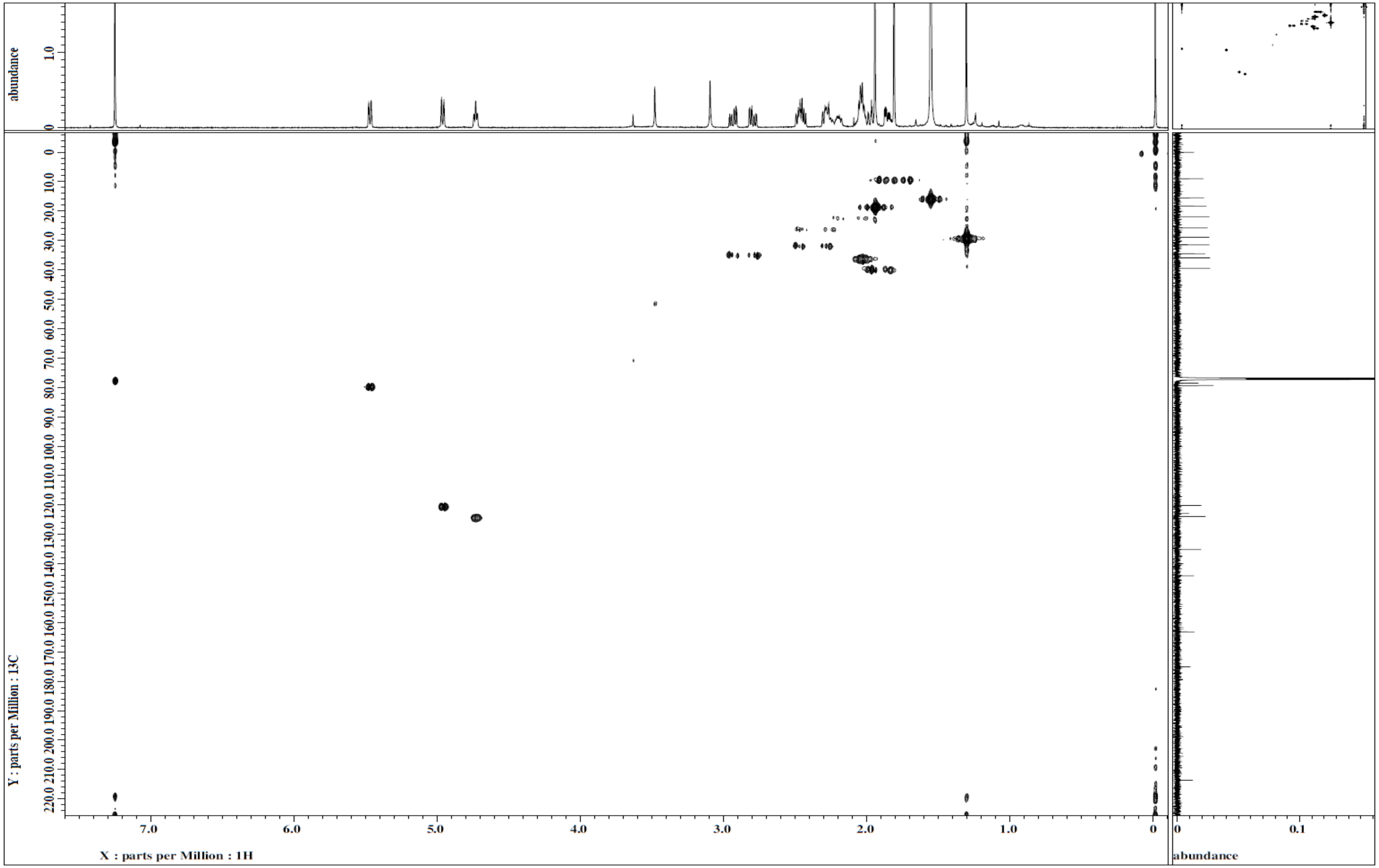

Figure S4. HMBC spectrum of 7 Keto, 8 hydroxy sarcophine (**1**) in $CDCl_3$.

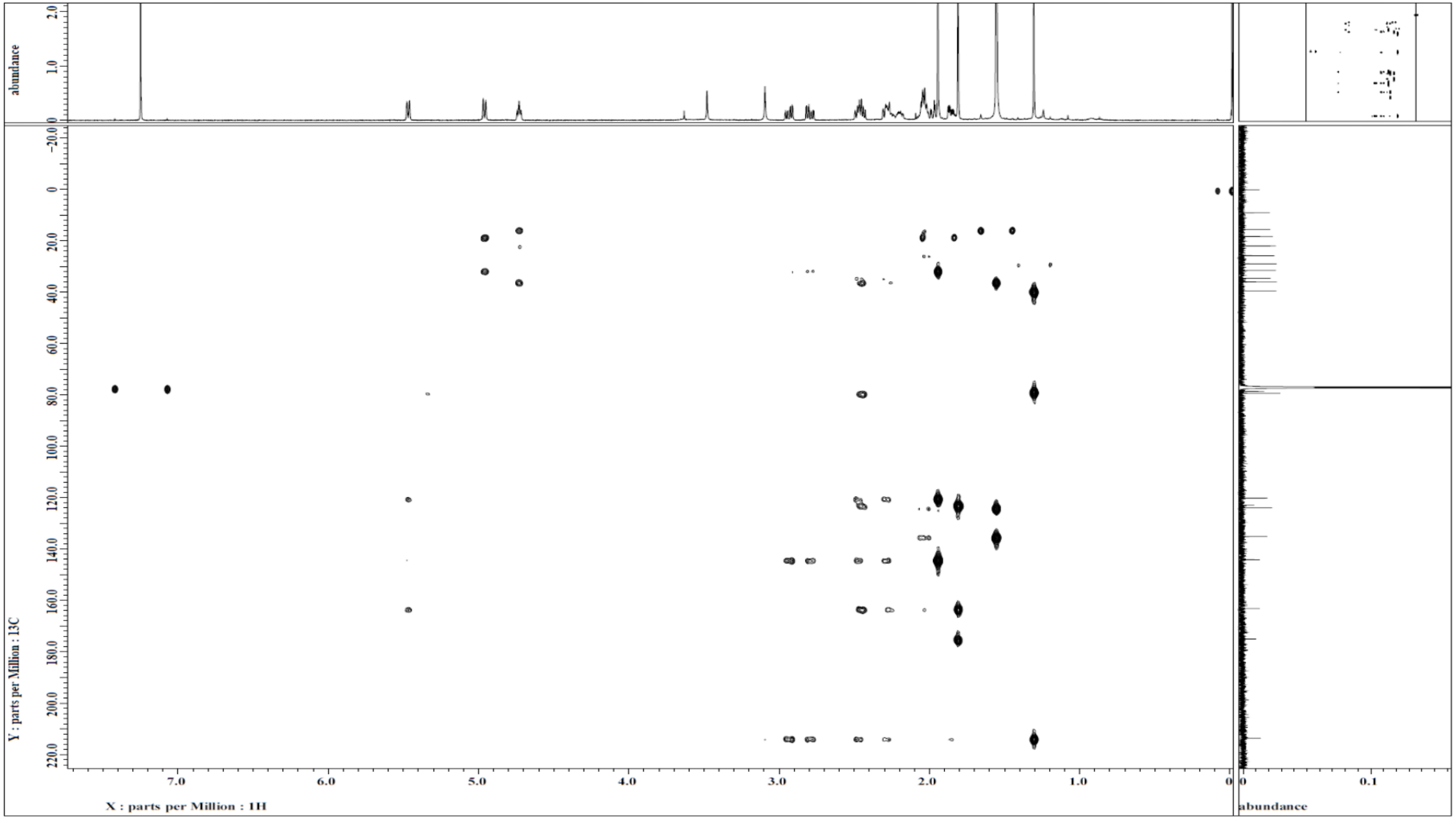

Figure S5. ^{1}H NMR spectrum of 7β-Chloro-8α-hydroxy-12-acetoxy-deepoxysarcophine (**2**) in $CDCl_3$.

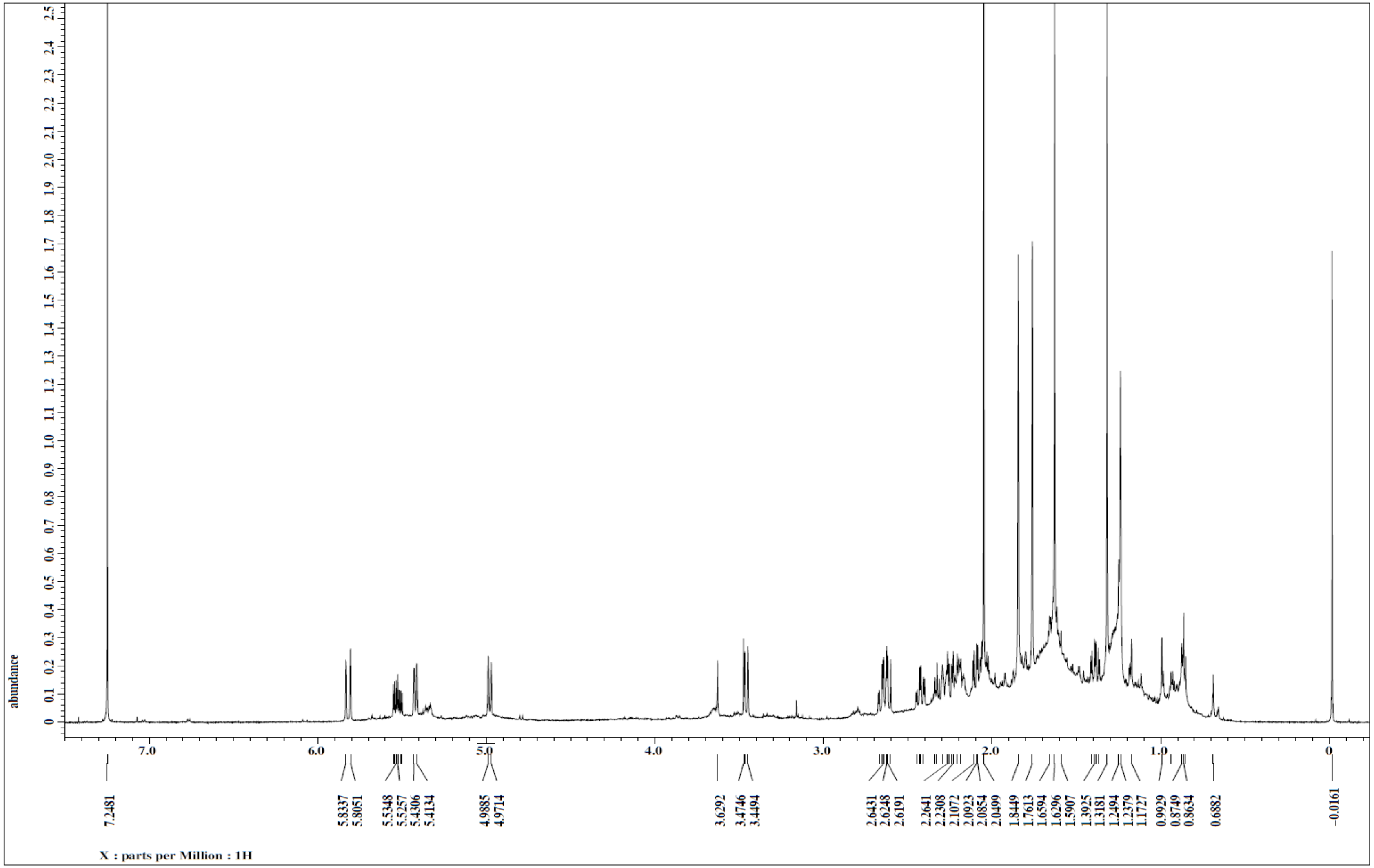

Figure S6. ^{13}C NMR spectrum of 7β-Chloro-8α-hydroxy-12-acetoxy-deepoxysarcophine (**2**) in $CDCl_3$.

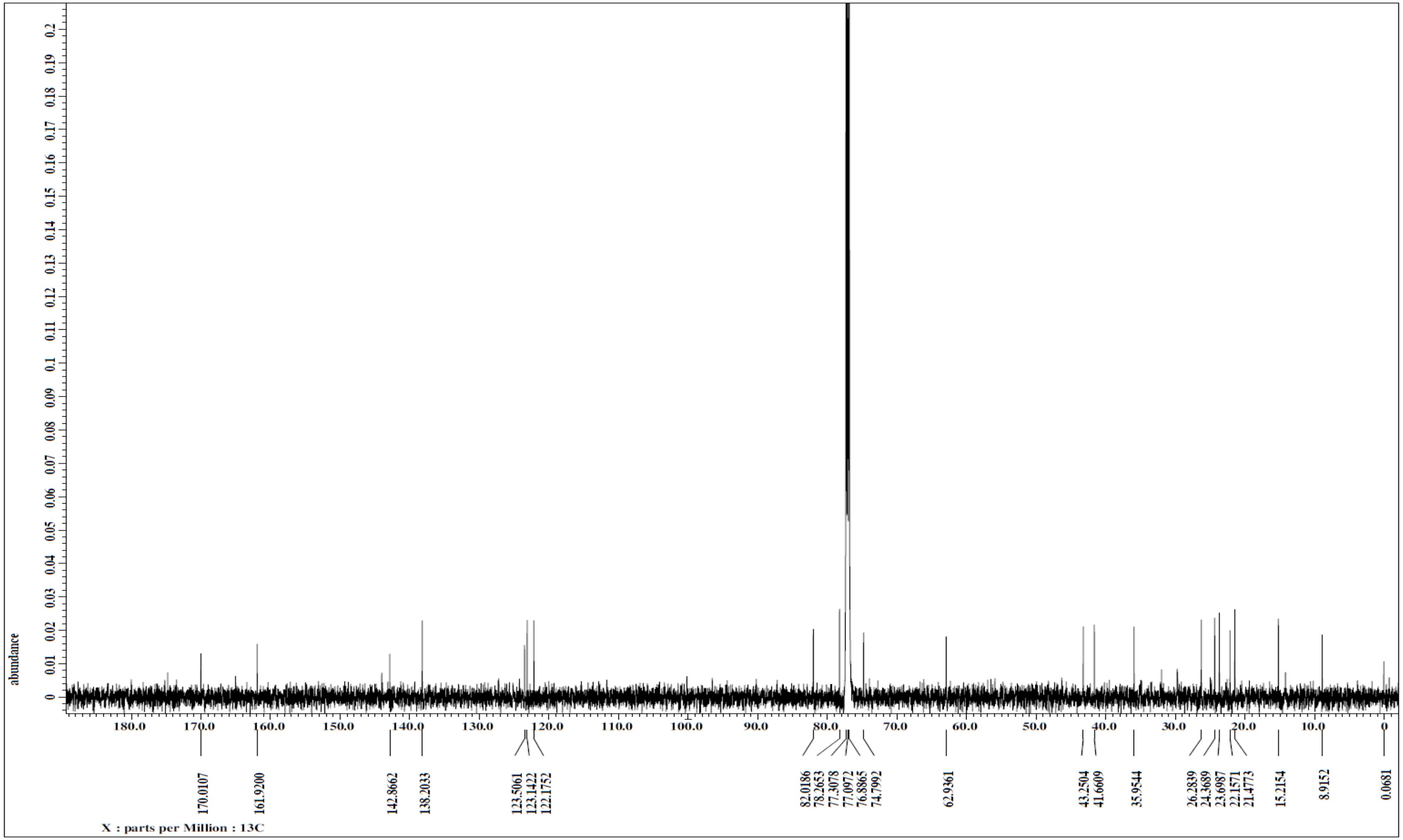

Figure S7. HMQC spectrum of 7β-Chloro-8α-hydroxy-12-acetoxy-deepoxysarcophine (**2**) in $CDCl_3$.

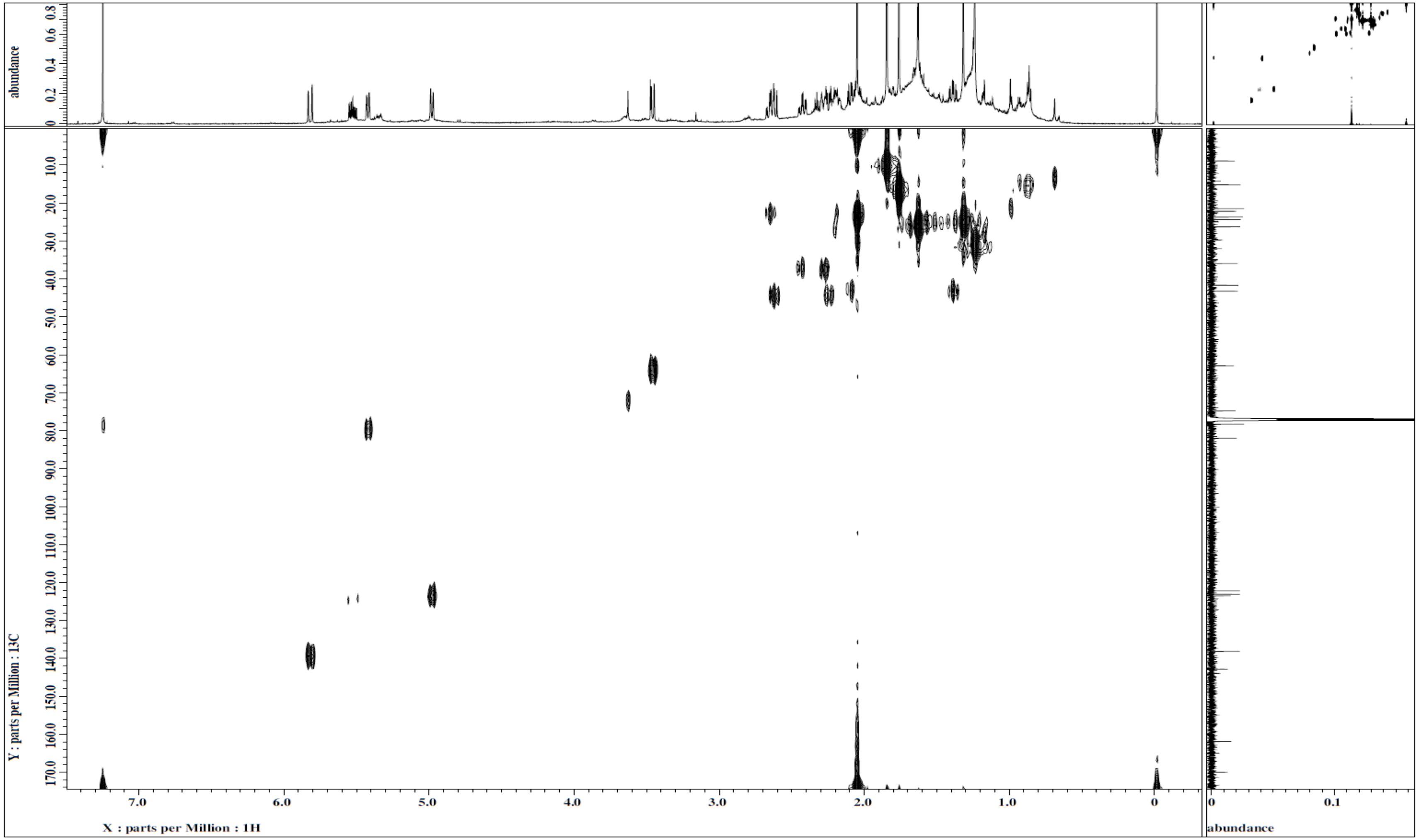

Figure S8. HMBC spectrum of 7β-Chloro-8α-hydroxy-12-acetoxy-deepoxysarcophine (**2**) in CDCl3.

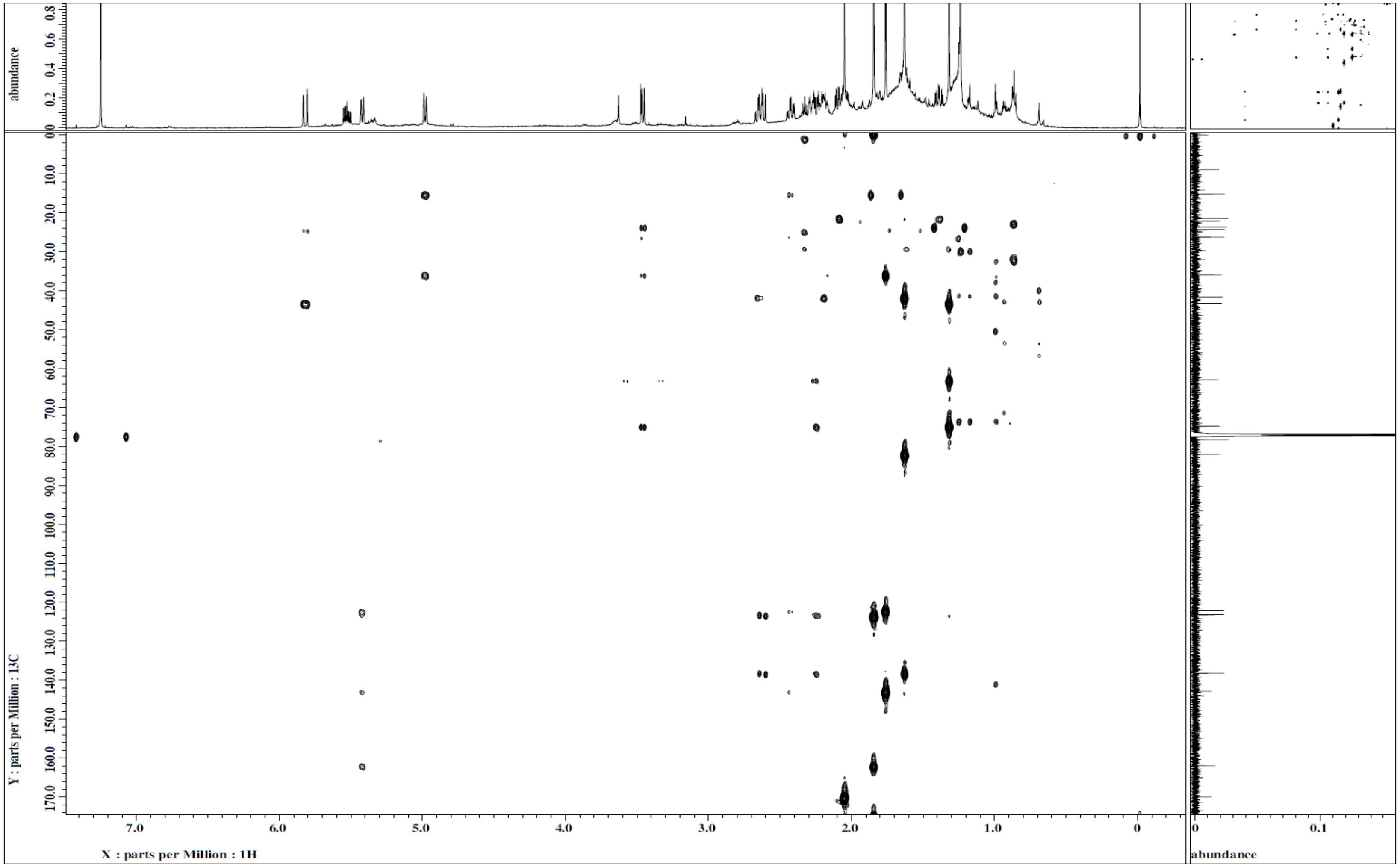

Figure S9. ^{1}H NMR spectrum of (*E*)-Methyl-3-(5-butyl-1-hydroxy-2,3-dimethyl-4-oxocyclopent-2-enyl)acrylate (**3**) in $CDCl_3$.

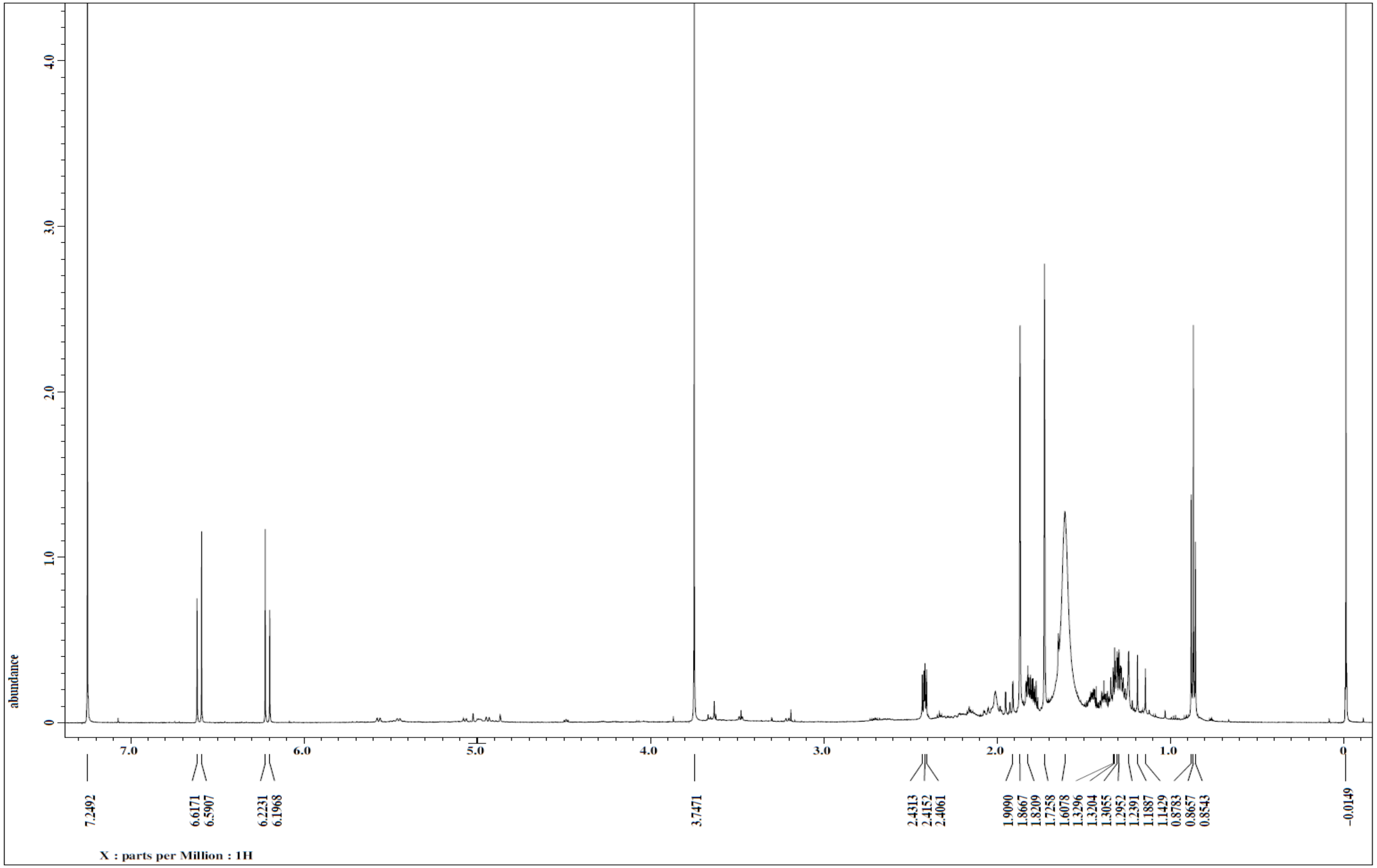

Figure S10. ^{13}C NMR spectrum of (*E*)-Methyl-3-(5-butyl-1-hydroxy-2,3-dimethyl-4-oxocyclopent-2-enyl)acrylate (**3**) in $CDCl_3$.

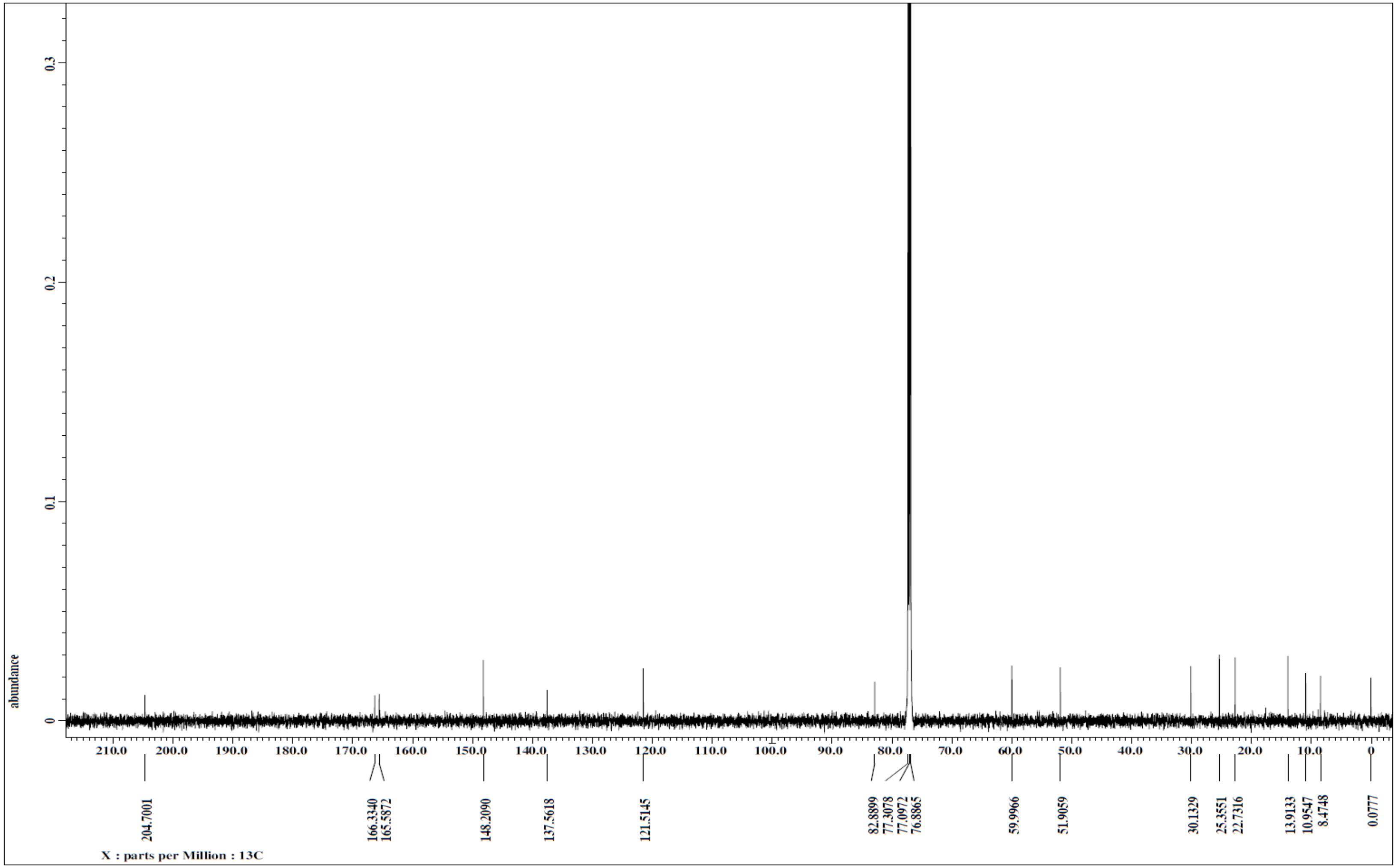

Figure S11. HMQC spectrum of (*E*)-Methyl-3-(5-butyl-1-hydroxy-2,3-dimethyl-4-oxocyclopent-2-enyl)acrylate (**3**) in $CDCl_3$.

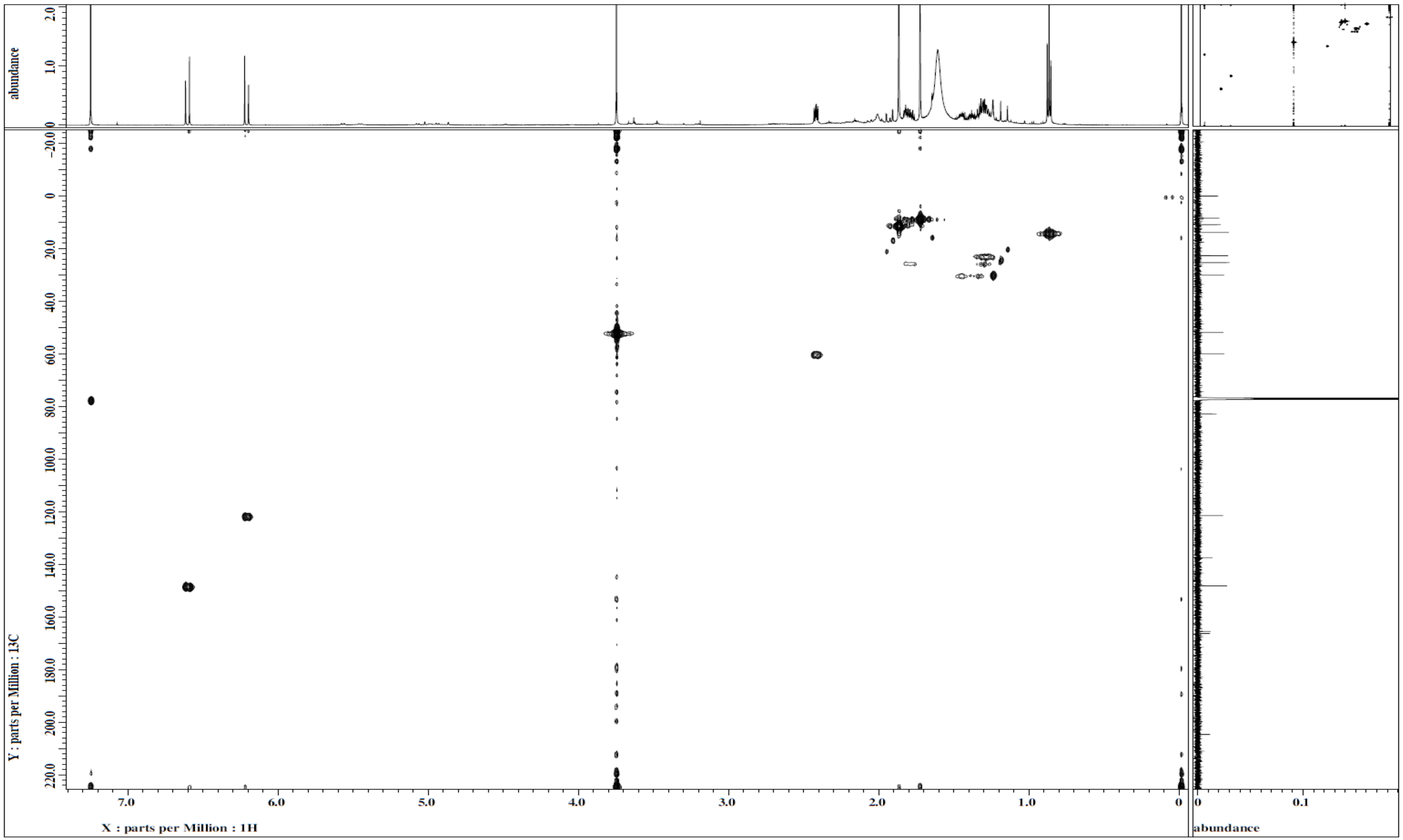

Figure S12. HMBC spectrum of (*E*)-Methyl-3-(5-butyl-1-hydroxy-2,3-dimethyl-4-oxocyclopent-2-enyl)acrylate (**3**) in $CDCl_3$.

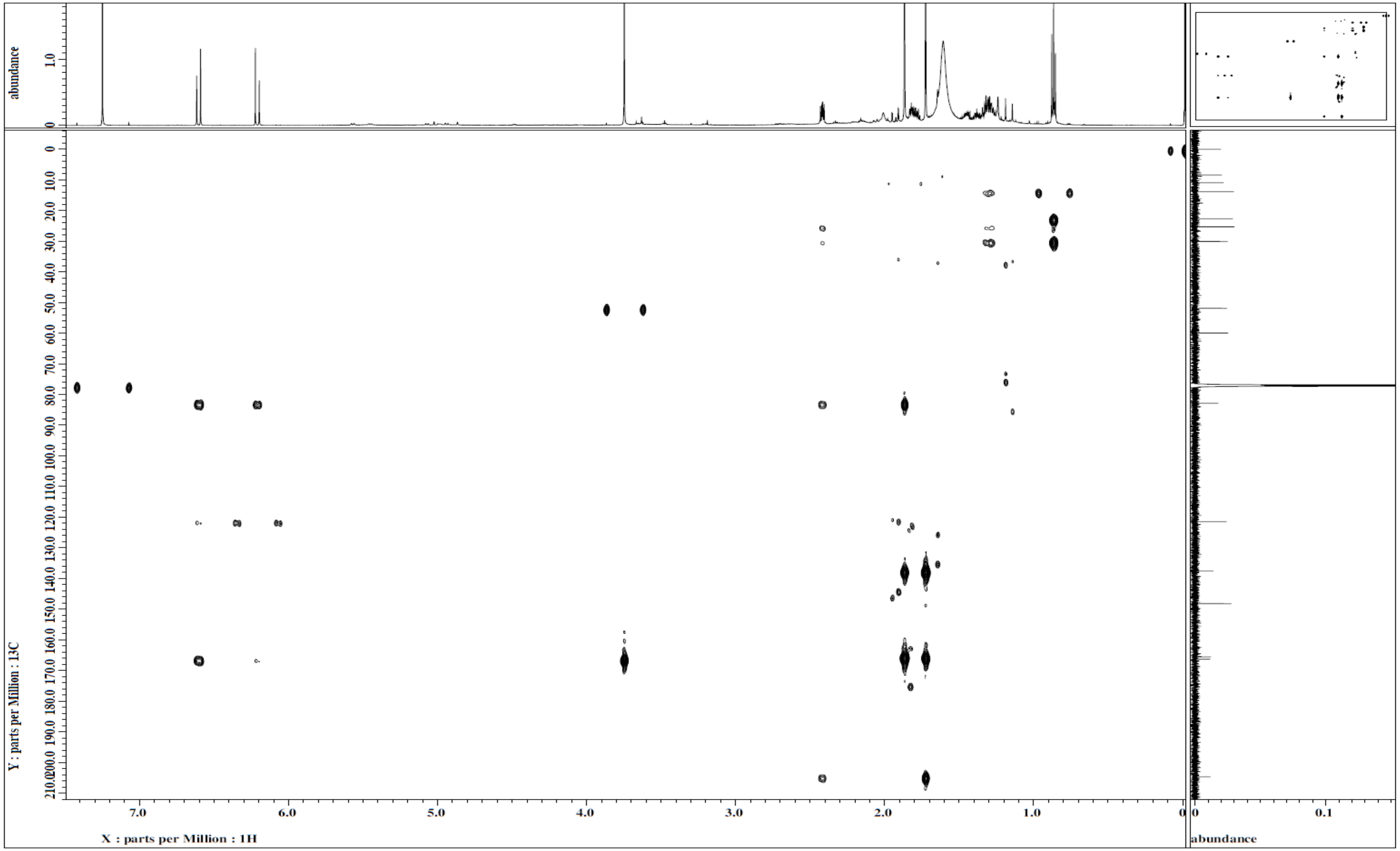

© 2014 by the authors; licensee MDPI, Basel, Switzerland. This article is an open access article distributed under the terms and conditions of the Creative Commons Attribution license (http://creativecommons.org/licenses/by/3.0/).
